# An imperfect test for a virus can Be worse than No test at all

**DOI:** 10.1002/hec.4254

**Published:** 2021-03-24

**Authors:** Mark Whitmeyer

**Affiliations:** ^1^ Hausdorff Center for Mathematics and Institute for Microeconomics University of Bonn Bonn Germany

**Keywords:** coronavirus, COVID‐19, group testing, information design

## Abstract

This note studies the effect of the availability of a test for a virus on the public health of a population. It is shown by example that the existence of a freely available and moderately informative test for a virus may lower society's welfare in comparison to the case where no test exists or access to the test is restricted. In this setting, any test provided to any subset of agents who would find it optimal not to isolate absent the test improves welfare.

## 1 INTRODUCTION

In 2020, the virus COVID‐19 swept through the globe. One particular difficulty presented by the virus is that infected individuals may be virtually asymptomatic and carry the virus without knowing it. Moreover, in the early stages of the pandemic, there were well‐publicized shortages of medical tests for the virus, which made it impossible to test everyone, even everyone with symptoms. To get around this, various solutions were proposed, including testing people in groups (Gollier & Gossner, 2020) and testing for inconclusive symptoms like a high temperature.^1^

This purpose of this note is to present a simple example that illustrates that the existence (and availability) of a moderately informative test can actually lower social welfare in comparison to the scenario when no such test exists (or is available). This analysis, thus,

1. Supports the regulation of costless tests;
2. Provides a word of caution against moderately informative tests; and
3. Emphasizes the importance of the choice as to whom should be tested.

In the model we explore, there is a heterogeneous population of agents with different exposure likelihoods. Each agent has a simple decision: whether to stay home and isolate (or self‐quarantine) or refrain from isolating and instead go out. An agent incurs a reward from not isolating, but possibly suffers a cost as well–she encounters others if out and neither wishes to become infected (if she is not infected) nor wishes to infect others (if she is infected). Thus, the prevalence of infected people who are not isolating is an endogenous equilibrium object determined by the individual isolation decisions of the agents.

Each agent's welfare is affected by the decisions of others, and crucially, a change in the infection rate of those out and about affects all of those who are not isolating. Accordingly, the mechanism that generates the possible welfare loss due to a test is the worsening of the participant pool. A false negative from a less than fully informative test can encourage risky people to refrain from isolating, increasing the chances of disease transmission. Although the information provided is itself valuable, the gain in welfare as a result of this information is outweighed by the increased disease prevalence among those an agent encounters.

The frequency of false negatives for the COVID‐19 virus is well‐documented. Indeed, the most common test for the virus, the reverse transcriptase polymerase chain reaction (RT‐PCR) test has false negative rates at initial presentation of symptoms that range from 30% to 40% (Ai et al., 2020; Fang et al., 2020; Yang et al., 2020). Moreover, these rates can vary considerably depending on the time since exposure (Kucirka et al., 2020). Computed Tomography (CT) scans may be more effective (Ai et al., 2020; Caruso et al., 2020; Fang et al., 2020), but even those may have high false negative rates in the first few days following the onset of symptoms (Kanne et al., 2020).

In this note, the agents are completely rational, yet can be made worse off by the availability of an imperfect test. This is because of the strategic nature of the societal interaction.

If this were merely a decision problem for each agent, any test would increase welfare.^2^ Moreover, it is understood that an imperfect test can encourage sub‐optimal behavior if people misunderstand or if people do not realize that the test is flawed. Here we discover that an imperfect test can be detrimental *even when its quality is common knowledge and when agents have no behavioral biases*.

Although we find that there are some tests and testing protocols that can lower welfare, Proposition 2.2 reveals that *any* test, provided it is given to people who have a (relatively) low likelihood of exposure–those who would refrain from isolating absent a test–is welfare improving, since it both provides those agents with more information but also ensures that the pool of agents participating in society improves. That is not to say that that group of people is the optimal group to test, merely that such a protocol, according to this framework, cannot reduce welfare.

The last statement leads us to the following caveat: in this paper, we do not attempt to characterize optimal testing protocols, nor do we provide a thorough cost‐benefit analysis of the participation/social‐distancing trade‐off. These are both worthwhile concerns, yet the goal of this paper is more modest. Instead, we merely wish to expose a counter‐intuitive aspect to testing, one that has seemingly been heretofore unmentioned and overlooked.

## 2 THE MODEL

Let us consider the following formal model. There is a population that consists of a continuum of agents with measure 1. The Bernoulli random variable, Θ, corresponds to the infection status of an agent, where Θ = 1 denotes that an agent is infected and Θ = 0 denotes that an agent is not infected. The only source of heterogeneity is an agent's prior exposure to the disease, which we term her type. That is, an agent's type is her likelihood of infection, $\mu \in \left[0,1\right]$, where $\mu :=P\left(\Theta =1\right)$. We impose that the population distribution of types has an atomless^3^ cumulative distribution function *F* with support on [0, 1].

Each agent has a simple choice: either isolate (action *I*) or participate (action *P*). If an agent isolates, she obtains a payoff normalized to 0. If an agent participates, then with probability *τ* she interacts with someone who is infected, where *τ* is the average infection likelihood of those participating. Hence, *τ* is the chance that a agent who is not isolating encounters an infected person. It is important to keep in mind that *τ* is an endogenous equilibrium object, determined by the isolation decisions of the agents in the populace.

If an agent of type *μ* participates then her payoff is

$$u\left(P;\mu ,\tau \right)=A-B\mu \left(1-\tau \right)-C\left(1-\mu \right)\tau$$

where *A* ≥ 0 is her reward from participating, *C* ≥ 0 is her loss from becoming infected, and *B* ≥ 0 is her loss from infecting someone else. Note that, in contrast to *τ*, *A*, *B*, and *C* are exogenous parameters.

The solution concept that we use is Nash Equilibrium: given the actions of the other agents, no agent has a (unilateral) profitable deviation. Due to the linearity of the payoff from *P* in an agent's type, any equilibrium must be of a particular cut‐off form: all agents whose types are above (or below) a certain threshold will isolate, and all those whose types are below (or above) that threshold will not. Because there is a coordination‐like aspect of the game, there may exist two kinds of equilibria; both those in which high types isolate and low types participate, but also the inverse. Throughout we restrict attention to the first class of equilibria, since it seems to correspond closest to people's behavior during the pandemic–by and large, sick people are encouraged to stay home, not go out. Moreover, the condition below ensures that such an equilibrium exists.

We begin by looking at the case in which there is no test, so that we can subsequently compare welfare to the case in which a test is available. We impose the following condition on the parameters:

Condition 2.1 There exists some type $\hat{\mu }$ such that

(i) $\begin{array}{cc} \;\;\;\;A+\left(\left(B+C\right)\hat{\tau }-B\right)\mu -C\hat{\tau } & \geq 0,\;\text{for}\;\text{all}\;\mu \leq \hat{\mu },\;\text{and}\\ A+\left(\left(B+C\right)\hat{\tau }-B\right)\mu -C\hat{\tau } & \leq 0,\;\text{for}\;\text{all}\;\mu \geq \hat{\mu }, \end{array}$

where,

$$\hat{\tau }=\tau \left(\hat{\mu }\right):=\frac{\int_{0}^{\hat{\mu }}xdF\left(x\right)}{F\left(\hat{\mu }\right)}$$

and

(ii) $\;\;\;\;\;\;\;\;\hat{\mu }\leq \frac{C}{B+C}$

$\hat{\tau }$ is a conditional expectation: it is the average infected likelihood of those participating (those whose type, *μ*, is less than the cutoff type, $\hat{\mu }$). Inequality (ii) ensures that in this equilibrium, the payoff of each type $\mu \in \left[0,\hat{\mu }\right]$ is decreasing in *τ*, which is realistic: the welfare of the participants gets worse as it becomes more likely that they encounter infected individuals.

A necessary condition for Condition 2.1 is that

$$\left(B+C\right)\hat{\tau }\leq B$$

That is, given $\hat{\tau }$, an agent's participation payoff is decreasing in her own type. If Condition 2.1 holds, then trivially there exists an equilibrium in which all types $\mu <\hat{\mu }$ participate and all types $\mu >\hat{\mu }$ isolate (indeed the first part of the condition is necessary and sufficient for such an equilibrium to exist). Denote the aggregate payoff from this equilibrium by *W*, *viz*.,

$$W:=\int_{0}^{\hat{\mu }}\left\{A+\left(\left(B+C\right)\hat{\tau }-B\right)x-C\;\hat{\tau }\right\}dF\left(x\right)$$

Clearly, this is an exceedingly simple model: the scenario is static and there are no benefits to testing other than to guide agents' isolation decisions. For instance, there is no contact tracing in this model, nor are there benefits to society from obtaining statistics about the spread, morbidity, or mortality of the disease. These are all important considerations for determining optimal testing policies.

Furthermore, outside of their infection likelihoods, agents in this model are homogeneous. We do not distinguish between essential and inessential workers, say, which is another vital component of a thorough cost‐benefit analysis of testing. The model's homogeneity in this dimension also does not allow us to tackle the subtle issue that, in reality, not all agents with the same infection likelihood are the same. Perhaps some high‐likelihood agents are travelers who have recently returned from a virus hot‐spot, whereas others are doctors, who are likely to be sick by virtue of their occupation. It is easy to see how distinguishing between these sorts of agents could be very important when choosing whom to test.^4^

Yet another simplification is that the agents who participate interact with each other randomly. They cannot choose the types with whom they interact, and neither the encounter rate of an agent nor whom an agent meets is affected by her infection probability. Again, this is perhaps unrealistic–a doctor is more likely to come into contact with infected individuals than a traveller who has returned home from a hot‐spot, yet our model does not allow for this distinction.

Naturally, a model intended to guide policy directly should include many, if not all, of these details that are conspicuously absent from this paper. However, as is noted above, the intent of this paper is not prescriptive. Our goal is to discover and understand the counterintuitive notion that testing may lower welfare. The model is kept deliberately simple in order to clearly illustrate the intuition of the result. We should expect similar incentives to be present in a more complicated model, but they might be hidden or obfuscated by other factors.

### 2.1 Testing participants cannot hurt

Now let us introduce testing to the scenario and derive our first result. Formally, a test, *π*, is a stochastic map:

$$\pi :\left\{0,1\right\}\to \Delta \left(S\right)$$

where *S* is some (compact) set of signal realizations. In the example that we explore later on, we assume that *S* consists merely of two signal realizations–a positive result and a negative result–but for now, we need not make such a restriction.

Then,

Proposition 2.2 *Let*  2.1 *hold*. *Then*, *any test*, *π*, *given to any subset of types in the interval* $\left[0,\hat{\mu }\right]$ *begets an equilibrium that yields society a payoff* that is *(at least weakly) greater than* *W*.

*Proof*. It suffices to show that the payoff of each type $\mu \in \left[0,\hat{\mu }\right]$ (weakly) increases in expectation. Moreover, recall that the second part of Condition 2.1 implies that the payoff of each type $\mu \in \left[0,\hat{\mu }\right]$ is decreasing in *τ*. Hence, if the pool of agents in society improves (*τ* decreases) the payoffs of those agents increase.

If the testing protocol begets an equilibrium in which the new average likelihood of participants (τ*) is equal to $\hat{\tau }$, then in expectation, the payoff of each type who is tested must weakly improve (this follows from Ramsey, 1990 and Blackwell, 1951). Naturally, this payoff is further improved if $\tau^{*}\leq \hat{\tau }$ and so we need only establish that there is an equilibrium in which all types $\mu \in \left[0,\mu^{*}\right]$, with $\mu^{*}\geq \hat{\mu }$ participate, and $\tau^{*}\leq \hat{\tau }$.

Let *G* be the new distribution over types (beliefs about infection likelihood) as a result of the test. Note that by definition,^5^ *G* is a mean‐preserving spread of *F*.^6^ There are two cases to consider: Case I, where $G\left(\hat{\mu }\right)=F\left(\hat{\mu }\right)$; and Case II, where $G\left(\hat{\mu }\right)<F\left(\hat{\mu }\right)$. The first case trivially yields the desired result (the pool of participants and the participation decisions of the agents are unchanged).

Let us consider Case II and search for an equilibrium in which all types $\mu \in \left[0,\mu^{*}\right]$ participate, where $\mu^{*}\geq \hat{\mu }$. It is straightforward to see footnote 7^7^ that

$$\hat{\tau }>\frac{\int_{0}^{\hat{\mu }}xdG\left(x\right)}{G\left(\hat{\mu }\right)}$$

Define

$$\tau \left(\mu \right):=\frac{\int_{0}^{\mu }xdG\left(x\right)}{G\left(\mu \right)}$$

which is obviously increasing in *μ*. Accordingly, since we have assumed that Condition 2.1 holds, there must exist some $\mu^{\prime }>\hat{\mu }$ such that $\tau \left(\mu^{\prime }\right)=\hat{\tau }$. Clearly,

$$\varphi \left(\mu \right):=A+\left(\left(B+C\right)\tau \left(\mu \right)-B\right)\mu -C\tau \left(\mu \right)$$

is continuous in *μ*. Evidently, $\varphi \left(\hat{\mu }\right)$ is positive and $\varphi \left(\mu^{\prime }\right)$ is negative, so by the intermediate value theorem there exists some $\mu^{*}\in \left(\hat{\mu },\mu^{\prime }\right)$, with $\varphi \left(\mu^{*}\right)=0$ and $\tau \left(\mu^{*}\right)<\hat{\tau }$. Consequently, there exists an equilibrium in which all types $\mu \in \left[0,\mu^{*}\right]$ participate, where $\mu^{*}>\hat{\mu }$ and $\tau^{*}:=\tau \left(\mu^{*}\right)<\hat{\tau }$.

Note that because belief is a martingale, for each agent that is tested, there must be some test result that ensures that her posterior type (remember, this is her posterior belief about her infection likelihood following a test result) is weakly less than $\hat{\mu }$ and hence μ*.

#### 2.1.1 An example

**FIGURE 1:**
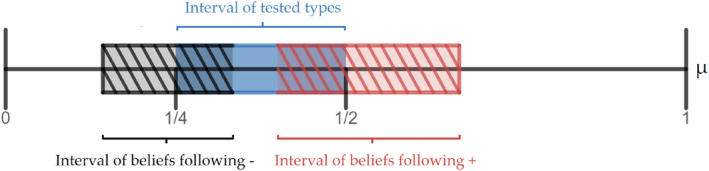
Example 2.1.1 Splitting of beliefs (types)

To add intuition to the proof, let us briefly explore an example. Namely, let *F* be the uniform distribution on $\left[0,1\right]$ and the parameters be such that $\hat{\mu }=\frac{1}{2}$. Consider a binary test, *π*, that is given to types $\mu \in \left[\frac{1}{4},\frac{1}{2}\right]$, with

$$\pi \left(+|1\right)=\pi \left(-|0\right)=\frac{2}{3}$$

where the set of test outcomes is $S=\left\{+,-\right\}$. Accordingly, each type in $\left[\frac{1}{4},\frac{1}{2}\right]$ will be “split” into two new types: with probability $\frac{\mu +1}{3}$, outcome + will realize and the posterior belief (new type) of agent *μ* will be $\frac{2\mu }{1+\mu }\in \left[\frac{2}{5},\frac{2}{3}\right]$; and with probability $\frac{2-\mu }{3}$, outcome − will realize and the posterior belief (new type) of agent *μ* will be $\frac{\mu }{2-\mu }\in \left[\frac{1}{7},\frac{1}{3}\right]$. This splitting is depicted in Figure 1.

**FIGURE 2:**
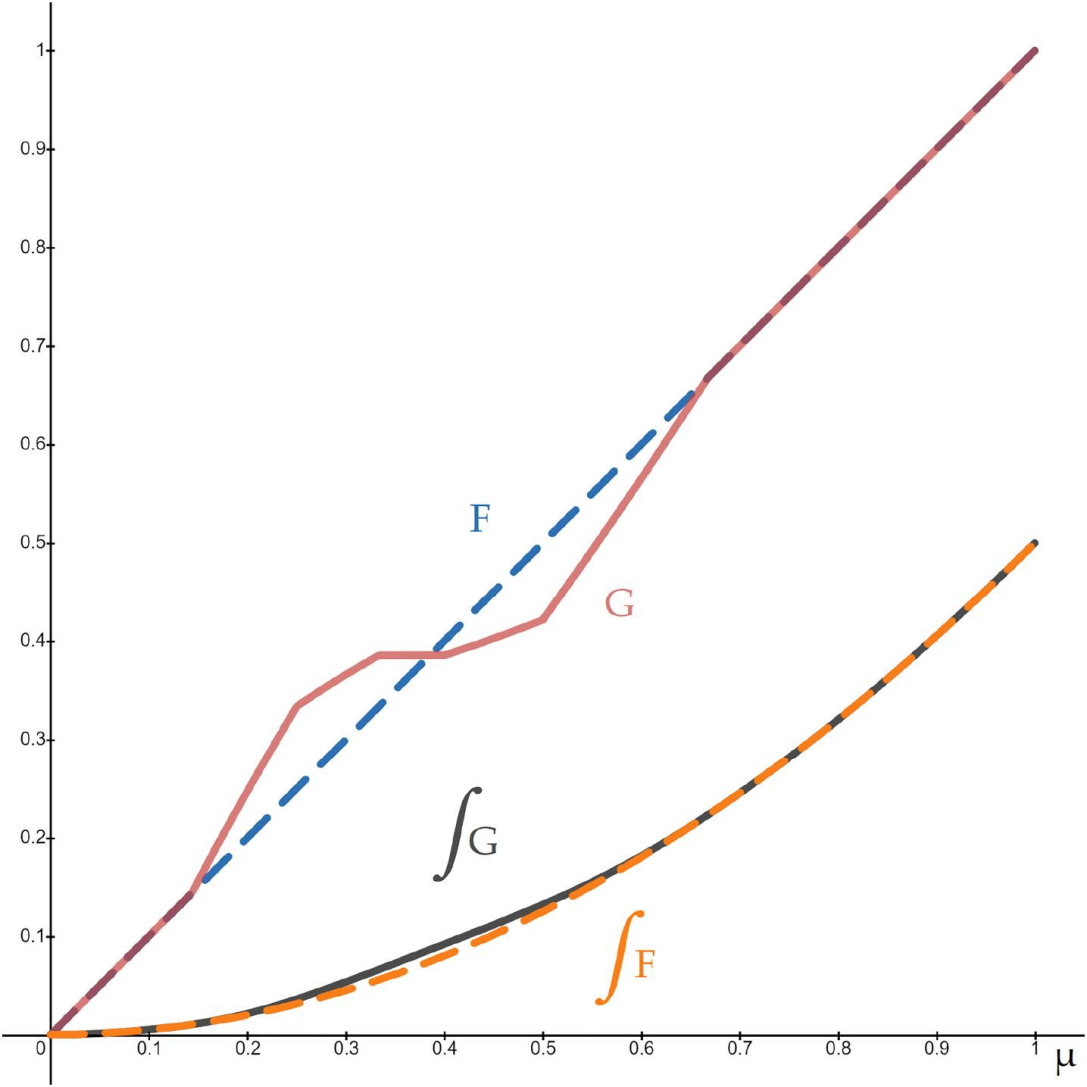
Example 2.1.1 Old (*F*, dashed lines) and new (*G*, solid lines) distributions of types

Furthermore, Figure 2 depicts the new cdf of types, *G*. Also depicted are $\int_{0}^{\mu }G\left(x\right)dx$ and $\int_{0}^{\mu }F\left(x\right)dx$, which illustrates that *G* is a mean‐preserving spread of *F*.

## 3 HOW TESTS CAN LOWER WELFARE

Let us turn our attention to a simplified version of the previous section's model. We reduce the model as follows: now, the population is inhabited by just two types of agents, *ω*_*H*_ (high likelihood) and *ω*_*L*_ (low likelihood). Proportion *q* = 3/4 of the population are *ω*_*L*_.

High likelihood agents, *ω*_*H*_, are infected with (prior) probability *μ*_*H*_, where

$$\mu_{H}:=P\left(\Theta =1|\omega_{H}\right)=5/8$$

Likewise, *μ*_*L*_ denotes the prior probability that type *ω*_*L*_ is infected:

$$\mu_{L}:=P\left(\Theta =1|\omega_{L}\right)=1/8$$

Recall that an agent may choose either to isolate (*I*) or not (*P*). We impose the following values for the parameters: *A*, her payoff from participation, equals 5/4; *B*, her penalty from infecting someone uninfected, is 2; and *C*, her penalty from becoming infected, is 4. Thus, if she has a belief *μ* that she is infected, her payoff from not isolating (*P*) is

$$u\left(P;\mu ,\tau \right)=\frac{5}{4}-2\mu \left(1-\tau \right)-4\left(1-\mu \right)\tau$$

which simplifies to

(1) $$u\left(P;\mu ,\tau \right)=\frac{5}{4}-4\tau -\left(2-6\tau \right)\mu$$

With no testing, the unique equilibrium is that in which agents of type *ω*_*H*_ isolate and agents of type *ω*_*L*_ participate. Since only agents of type *ω*_*L*_ are participating, the likelihood that an agent who is not isolating is infected is merely the likelihood that an agent of type *ω*_*L*_ is infected; *viz*., *τ* = *μ*_*L*_ = 1/8. On path, an agent of type *ω*_*L*_ obtains a payoff of 19/32, which is bigger than 0, her payoff from isolating. Should an agent of type *ω*_*H*_ deviate and participate, she would obtain a payoff of −1/32, less than her isolation payoff of 0.

In fact, an agent of type *ω*_*H*_ would always prefer to isolate (since *μ*_*H*_ is so high), unless *τ* were precisely 0, which could never happen at equilibrium. Consequently, this equilibrium is unique.

Without testing, the aggregate welfare for society, *V*, is

$$V=q\left(\frac{5}{4}-4\tau -\left(2-6\tau \right)\mu_{L}\right)=\frac{3}{4}\left(\frac{19}{32}\right)=\frac{57}{128}\approx .45$$

### 3.1 After the introduction of a test

We introduce a binary test to the scenario. The set of signal realizations is $S:=\left\{+,-\right\}$, corresponding to a positive test and a negative test, respectively. Consequently, *π* can be written in terms of the variable *p*, where

$$p:=\pi \left(-|1\right),\;\text{and}\;\pi \left(-|0\right)=1$$

The situation can be conveniently described by the following joint distribution of *S* and Θ for an agent with prior *μ*_*i*_, *i* = *H*, *L*:

**Table:** 

Θ \ *S*	−	+	$P\left(\Theta \right)$
1	*μ*_*i*_ *p*	$\mu_{i}\left(1-p\right)$	*μ*_*i*_
0	$\left(1-\mu_{i}\right)$	0	1 − *μ*_*i*_
$P\left(S\right)$	$\mu_{i}p+\left(1-\mu_{i}\right)$	$\mu_{i}\left(1-p\right)$	1

Note that in this model there are no false positives. This is done for expositional convenience and to allow us to focus on the effect of the high false negative rates noted in the introduction.

Next, we look at the Nash Equilibria of the participation game with testing. As in Section 2, we focus on equilibria in which agents who think their infection likelihood is low participate and agents who think their infection likelihood is high do not. In this example, absent a test, this is the only equilibrium that exists, and we suppose that this is still the equilibrium selected following a test. To put another way, we assume that introduction of the test does not qualitatively alter the equilibrium selected to an (ostensibly less realistic) equilibrium in which low types isolate and high types participate.

We consider three cases: in the first, only agents of type *ω*_*H*_ have access to the test.

#### 3.1.1 Case 1 (testing only high likelihood)

Here we suppose that only agents of type *ω*_*H*_ may take the test. There are three regions of false negative probabilities, *p*, each of which beget a different equilibrium. If *p* is sufficiently low (*p* ≤ 0.73), then agents of type *ω*_*L*_ participate and agents of type *ω*_*H*_ participate if and only if they get a negative test result. On the other hand, if *p* is in an intermediate range (0.9 ≥ *p* ≥ 0.73), then such an equilibrium no longer exists. All agents of type *ω*_*L*_ continue to participate, but now only a fraction of the types *ω*_*H*_ who have received negative results participate. Finally, if *p* is too high, only agents of type *ω*_*L*_ participate.

We start by calculating the values of *p* such that at equilibrium agents of type *ω*_*L*_ participate and agents of type *ω*_*H*_ participate if and only if they get a negative test result.

The crucial variable is *τ*, the likelihood of encountering an infected agent while participating. Using the law of total probability, it is

$$\tau_{p}^{H}=\frac{q\mu_{L}+\left(1-q\right)\mu_{H}p}{q+\left(1-q\right)\left(\mu_{H}p+1-\mu_{H}\right)}=\frac{5p+3}{5p+27}$$

Using Bayes' law, the probability that type *ω*_*H*_ is infected after a negative test is

(2) $$\mu_{H}^{-}=\frac{\mu_{H}p}{\mu_{H}p+1-\mu_{H}}=\frac{5p}{5p+3}$$

Then, using Expression 1, agents of type *ω*_*H*_ will participate after − if and only if

(3) $$\frac{5}{4}-4\tau_{p}^{H}-\left(2-6\tau_{p}^{H}\right)\mu_{H}^{-}\geq 0$$

which simplifies to $p\leq \frac{9}{5}-\frac{12}{5\sqrt{5}}\approx .73$.

It is easy to verify that agents of type *ω*_*L*_ prefer to participate for this range of *p*. Moreover, clearly, agents of type *ω*_*H*_ have no profitable deviation to action *P* after + , since they are sure that they are infected.

Should Inequality 1 fail to hold, there is no longer an equilibrium in which all agents of type *ω*_*H*_ who have obtained a negative test participate. However, for moderate *p*, there is also no equilibrium in which *none* of those agents participate, since the resulting *τ* would be low enough to entice participation. Instead, fraction *σ* of the agents of type *ω*_*H*_ who have seen a negative test participate.

Observe that $\mu_{H}^{-}$ is the same as above, but the likelihood of encountering an infected agent is shaped by *σ*. This is

(4) $$\tau \left(\sigma \right)=\frac{q\mu_{L}+\left(1-q\right)\sigma \mu_{H}p}{q+\left(1-q\right)\sigma \left(\mu_{H}p+1-\mu_{H}\right)}=\frac{5p\sigma +3}{5p\sigma +3\sigma +24}$$

Because some of the agents of type *ω*_*H*_ isolate after a negative result and others do not, we need them to be indifferent as to whether they participate after −. Thus, using Expression 1,

$$\frac{5}{4}-4\tau \left(\sigma^{*}\right)-\left(2-6\tau \left(\sigma^{*}\right)\right)\mu_{H}^{-}=0$$

Substituting in for $\tau \left(\sigma^{*}\right)$, we obtain

$$\sigma^{*}=\frac{24\left(10p-9\right)}{5\left(25p^{2}-42p+9\right)}$$

which is feasible (lies in the interval [0, 1]) provided 0.73 ≤ *p* ≤ 0.9. Substituting σ* into Equation 4 we obtain the equilibrium infection likelihood

(5) $$\tau \left(\sigma^{*}\right)=\frac{15\left(1-p\right)}{48-40p}$$

If *p* ≥ 0.9, then the only equilibrium yields the same payoff as the scenario without testing. The test is too uninformative to persuade any agents of type *ω*_*H*_ and so as in the case without testing, only agents of type *ω*_*L*_ participate, yielding a payoff of 0.45.

We finish the analysis of Case 1 by inspecting aggregate welfare as a function of the probability *p*, *V* ^*H*^ (*H* for “High likelihood”), the details of whose derivation we leave to Appendix A.1:

$$V^{H}=\left\{\begin{array}{cc} \frac{125p^{2}-1530p+1917}{128\left(5p+27\right)},\;\; & 0\leq p\leq \frac{9}{5}-\frac{12}{5\sqrt{5}}\\ -\frac{105p-9}{640p-768},\;\; & \frac{9}{5}-\frac{12}{5\sqrt{5}}\leq p\leq \frac{9}{10}\\ \frac{57}{128},\;\; & \frac{9}{10}\leq p\leq 1 \end{array}\right.$$

Comparing this to *V*, aggregate welfare when there are no tests, we see that *V* ^*H*^ < *V* = 57/128 for $p\in \left(.21,.9\right)$, and *V* ^*H*^ = *V* for *p* ∈ [0.9, 1]. Moreover, for all $p\in \left[.73,.9\right]$, there is a Pareto decrease in welfare: agents of type *ω*_*H*_ are no better off and agents of type *ω*_*L*_ are strictly worse off.

#### 3.1.2 Case 2 (testing only low likelihood)

What if those tests from Case 1 were instead given to agents of type *ω*_*L*_? To ensure a fair comparison, suppose that there are only enough tests to serve measure 1/4 of agents (so only 1/3 of agents of type *ω*_*L*_ get tested). At equilibrium, untested agents of type *ω*_*L*_ participate, tested agents of type *ω*_*L*_ participate if and only if they have a negative test result, and agents of type *ω*_*H*_ do not participate.

Now,

$$\tau_{p}^{L}=\frac{\frac{2q}{3}+\frac{q}{3}p}{\frac{2q}{3}+\frac{q}{3}\left(\mu_{L}p+1-\mu_{L}\right)}\mu_{L}=\frac{p+2}{p+23}$$

It is obvious that agents of type *ω*_*L*_ participate after a negative test since they would even with a perfectly uninformative test (*p* = 1). Likewise, they do not participate after a positive test since they are sure that they are infected. Agents of type *ω*_*H*_ do not participate since $\tau_{p}^{L}$ is strictly greater than 0.

Consequently, aggregate welfare as a function of *p* is *V* ^*L*^ (*L* for “Low likelihood”):

$$\begin{array}{ll} V^{L} & =\frac{2q}{3}\left(\frac{5}{4}-4\tau_{p}^{L}-\left(2-6\tau_{p}^{L}\right)\mu_{L}\right)\\ & \;+\frac{q}{3}\left(\mu_{L}p+1-\mu_{L}\right)\left(\frac{5}{4}-4\tau_{p}^{L}-\left(2-6\tau_{p}^{L}\right)\mu_{L}^{-}\right)\\ & =\frac{5p^{2}-274p+1637}{128\left(p+23\right)} \end{array}$$

where $\mu_{L}^{-}$, the probability that type *ω*_*L*_, is infected after a negative test is obtained using Bayes' law. Evidently, *V* ^*L*^ ≥ *V* for all *p*.

This case yields precisely the result that we uncovered in Section 2.1. Namely, any test applied to any subset of the population whose members are participating in the absence of a test must improve society's welfare.

#### 3.1.3 Case 3 (testing everyone)

What if both types of agents have access to the test? Perhaps unsurprisingly, this case is qualitatively identical to Case 1. For a sufficiently low *p* (*p* ≤ 0.76), agents of type *ω*_*L*_ participate and agents of type *ω*_*H*_ participate if and only if they get a negative test result. If *p* is in an intermediate range (0.91 ≥ *p* ≥ 0.76), then all agents of type *ω*_*L*_ continue to participate, and only a fraction of the types *ω*_*H*_ who have received negative results participate. If *p* is too high, only agents of type *ω*_*L*_ participate.

Leaving its derivation to Appendix A.1–since it is identical to the work for Case 1 *mutatis mutandis*–aggregate welfare as a function of *p* is *V* ^*T*^:

$$V^{T}=\left\{\begin{array}{cc} \frac{5p^{2}-42p+45}{16\left(p+3\right)},\;\; & 0\leq p\leq \frac{39-6\sqrt{11}}{25}\\ \frac{3p}{24-20p},\;\; & \frac{39-6\sqrt{11}}{25}\leq p\leq \frac{69-2\sqrt{534}}{25}\\ \frac{15p^{2}-294p+735}{128p+896},\;\; & \frac{69-2\sqrt{534}}{25}\leq p\leq 1 \end{array}\right.$$

Evidently, *V* ^*T*^ ≤ *V* for $p\in \left[.51,.9\right]$. As in Case 1, there is an interval of *p* values, [0.76,.9], that begets a Pareto decrease in the equilibrium welfare for society. Curiously, Case 3 illustrates that an extremely uninformative test can be strictly welfare improving. It provides some information to agents of type *ω*_*L*_ but is insufficiently informative to persuade any agents of type *ω*_*H*_ to participate and so does not worsen the participant pool.

Unsurprisingly, welfare for society is higher when everyone can get tested (Case 3), than when only agents of type *ω*_*H*_ can get tested (Case 1). In both cases, an increase in *τ* drives the decrease in welfare, but in the Case 3, the effect of this increase in *τ* is not as pernicious, due to the information acquired by the low likelihood types.

**FIGURE 3:**
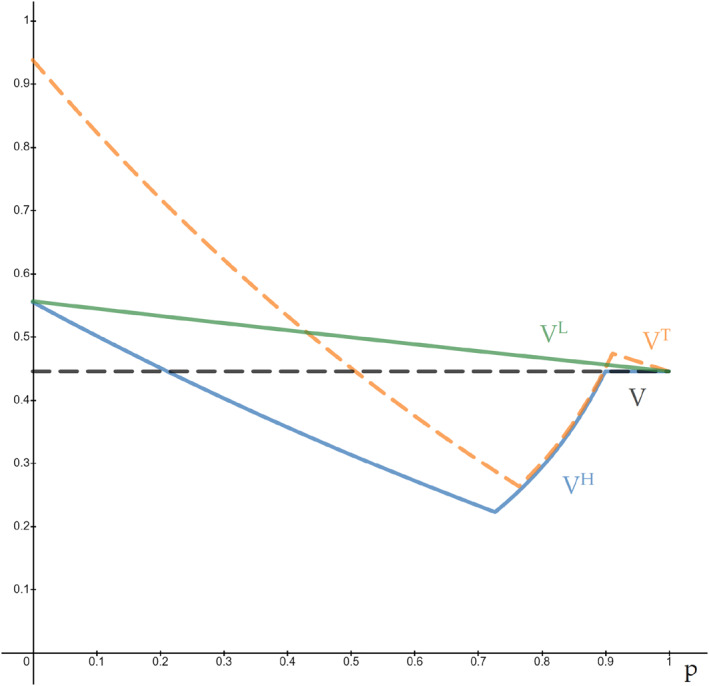
Society's welfare in Cases 1 (*V* ^*H*^, solid line), 2 (*V* ^*L*^, solid line), 3 (*V* ^*T*^, dashed line), *&* with no testing (*V*, dashed line)

A graph of society's welfare as a function of *p* when there is no testing and in each of the three cases is depicted in Figure 3.

Finally, recall that in the example, the test does not generate false positives. How does the false positive rate affect welfare? As it turns out, the effect of the false positive rate is also ambiguous. It is possible that a high false positive rate could be better for society due to its effect on the isolation decisions of agents of type *ω*_*H*_. Naturally, there are also regions of the parameters in which welfare is decreasing in the false positive rate, which is driven by the intrinsic value of information.

## 4 BRIEF DISCUSSION

Determining the optimal way to guide a society through a pandemic and calculating optimal testing protocols is a challenging task. Such an undertaking is made even more difficult by the myriad of constraints that must be satisfied–logistical, resource, cognitive, and political, to name a few. Here we bypass such concerns and merely explore a counter‐intuitive aspect of testing engendered by the interconnected nature of society.

Namely, we find that the introduction of a moderately informative test can make society worse off and can even result in a Pareto decrease in welfare. This is because the test worsens the pool of participants–it makes it more likely that agents encounter infected agents who, thanks to the test, are sufficiently confident that they are not infected due to a false negative produced by the test.

On the other hand, as Proposition 2.2 illustrates, every test is welfare improving, so long as it is given (or available) only to agents who participate in equilibrium in the absence of the test. Such a protocol guarantees that the pool of participants in society improves and provides valuable information to agents, to boot.

As we have stressed throughout, this paper does not prescribe optimal testing protocols. There are a number of contemporaneous papers that do, under a variety of different assumptions and set‐ups. Kasy and Teytelboym (2020) allow for perfectly informative (but costly) tests, and investigate the dynamic problem of how to test individuals in order to both inform quarantine protocols and also learn about the virus's prevalence rate. Ely et al. (2020) assume a finite quantity of heterogeneous tests and study how to allocate such tests to heterogeneous agents. Lipnowski and Ravid (2020) explore a similar problem, but look at how to *pool* tests optimally. Deb et al. (2020), in turn, allow for targeted testing *and* transfers to agents.

Crucially, in comparison to this paper, none of those papers contain a strategic interaction between the agents in the population. In each such paper, agents and planners are merely tasked with decision problems and so the value of information is positive. The important decision then, is the choice of whom to test or how to test, given limited resources or costly tests. As we encounter here, introducing strategic concerns adds a novel wrinkle, one that may be important to keep in mind when determining policy.

Our discovery that information may be detrimental is related to the phenomenon discovered by Kremer (1996), who shows that increased prevalence of the AIDS virus may worsen the pool of available partners due to the fatalism of high‐activity people. In this note, the welfare loss is also due to a worsened pool of participants, which effect is driven by not by fatalism but by the increased (rational) confidence of high‐likelihood individuals consequent to negative test results.

A number of other recent papers have pointed out other counterintuitive incentives in models of epidemics and disease transmission. Those include Talamàs and Vohra (2020), who show that the introduction of a moderately effective vaccine can result in Pareto losses in welfare for society; and Heinsalu (2020), who illustrates that increasing the infection risk early in a pandemic may be optimal. However, the mechanism behind the result in Talamàs and Vohra (2020) is completely different to that in this paper. There, the authors assume a network structure and allow agents to choose with whom they match. In contrast to this paper, fixing the partnership structure of their model or imposing that matching is random ensures that even a partially effective vaccine is a Pareto improvement to welfare. In their paper, it is only when agents may choose with whom to match that imperfect vaccines can have a destabilizing effect on society's partnership network, begetting a denser (and hence worse) network, and thereby lowering welfare.

With that in mind, it would be interesting to investigate whether the results of this paper extend to a finite population with a network structure. Likewise, it is unclear how allowing agents to make more sophisticated decisions–letting them choose not just whether to isolate but also with whom to interact–would affect the findings. Given the results of Talamàs and Vohra (2020), it is reasonable to suspect that a similar destabilizing effect could manifest as the result of an imperfect test.

## CONFLICT OF INTEREST STATEMENT

I, Mark Whitmeyer, declare no conflict of interest.

5

## Data Availability

Data sharing not applicable—no new data generated, or the article describes entirely theoretical research.

## ENDNOTES

^1^ See, e.g., this April 4th CNBC article.

^2^ See e.g. Ramsey (1990); Blackwell (1951), who (among many others) establish that the well‐known result that in decision problems, the value of information is always positive.

^3^ This assumption is not significant, but eases notation.

^4^ I thank an anonymous referee for highlighting this aspect of optimal testing.

^5^ Indeed, this is precisely the definition of a mean‐preserving spread introduced in Rothschild and Stiglitz (1970).

^6^ Moreover, since *F* is atomless, *G* will not have atoms except possibly at 0 and 1. The proof proceeds with the implicit assumption that *G* does not have an atom on 1, but that is just to ease notation and simplify the arguments. If *G* does have an atom there, the result continues to hold.

^7^ Refer to Lemma A.1 in the Appendix.

## APPENDIX A A | Omitted Results and Derivations

### A.1

Lemma A.1

$$\hat{\tau }=\frac{\int_{0}^{\hat{\mu }}xdF\left(x\right)}{F\left(\hat{\mu }\right)}>\frac{\int_{0}^{\hat{\mu }}xdG\left(x\right)}{G\left(\hat{\mu }\right)}$$

*Proof*. Directly, using integration by parts

$$\frac{\int_{0}^{\hat{\mu }}xdF\left(x\right)}{F\left(\hat{\mu }\right)}=\hat{\mu }-\frac{\int_{0}^{\hat{\mu }}F\left(x\right)dx}{F\left(\hat{\mu }\right)}>\hat{\mu }-\frac{\int_{0}^{\hat{\mu }}F\left(x\right)dx}{G\left(\hat{\mu }\right)}>\hat{\mu }-\frac{\int_{0}^{\hat{\mu }}G\left(x\right)dx}{G\left(\hat{\mu }\right)}=\frac{\int_{0}^{\hat{\mu }}xdG\left(x\right)}{G\left(\hat{\mu }\right)}$$

which used the facts that $F\left(\hat{\mu }\right)>G\left(\hat{\mu }\right)$ and *G* is a mean preserving spread of *F*, of which one definition is that

$$\int_{0}^{\mu }G\left(x\right)dx\geq \int_{0}^{\mu }F\left(x\right)dx$$

for all $\mu \in \left[0,1\right]$ (an equivalent statement is that *F* second order stochastically dominates *G*).

### A.1 Sections 3.1.1 and 3.1.3 aggregate welfare derivations

Case 2 (Section 3.1.2) is omitted, since the lone derivation may be found in the text.

### A.1.1 Case 1 (testing only high likelihood)

For $0\leq p\leq \frac{9}{5}-\frac{12}{5\sqrt{5}}\approx .73$, aggregate welfare, *V* ^*H*^, is

$$\begin{array}{cc} V^{H} & =q\left(\frac{5}{4}-4\tau_{p}^{H}-\left(2-6\tau_{p}^{H}\right)\mu_{L}\right)+\left(1-q\right)\left(\mu_{H}p+1-\mu_{H}\right)\left(\frac{5}{4}-4\tau_{p}^{H}-\left(2-6\tau_{p}^{H}\right)\mu_{H}^{-}\right)\\ & =\frac{125p^{2}-1530p+1917}{128\left(5p+27\right)} \end{array}$$

For $\frac{9}{10}\geq p\geq \frac{9}{5}-\frac{12}{5\sqrt{5}}$, since each high type, *ω*_*H*_ is receiving a payoff of 0, we have

$$V^{H}=q\left(\frac{5}{4}-4\tau \left(\sigma^{*}\right)-\left(2-6\tau \left(\sigma^{*}\right)\right)\mu_{L}\right)=-\frac{105p-9}{640p-768}$$

If $1\geq p\geq \frac{9}{10}$, aggregate welfare is the same as in the case without a test; namely, $V^{H}=\frac{57}{128}\approx .45$.

### A.1.2 Case 3 (testing everyone)

First, let $0\leq p\leq \frac{39-6\sqrt{11}}{25}\approx .76$. Let us calculate $\tau_{p}^{T}$. Directly,

$$\tau_{p}^{T}=p\frac{q\mu_{L}+\left(1-q\right)\mu_{H}}{q\left(\mu_{L}p+1-\mu_{L}\right)+\left(1-q\right)\left(\mu_{H}p+1-\mu_{H}\right)}=\frac{p}{p+3}$$

Then, aggregate welfare, *V* ^*T*^, is

$$\begin{array}{cc} V^{T}=q\left(\mu_{L}p+1-\mu_{L}\right) & \left(\frac{5}{4}-4\tau_{p}^{T}-\left(2-6\tau_{p}^{T}\right)\mu_{L}^{-}\right)\\ & +\left(1-q\right)\left(\mu_{H}p+1-\mu_{H}\right)\left(\frac{5}{4}-4\tau_{p}^{T}-\left(2-6\tau_{p}^{T}\right)\mu_{H}^{-}\right) \end{array}$$

which simplifies to

$$V^{T}=\frac{5p^{2}-42p+45}{16\left(p+3\right)}$$

For $\frac{39-6\sqrt{11}}{25}\leq p\leq \frac{69-2\sqrt{534}}{25}\approx .91$, we have

$$V^{T}=q\left(\mu_{L}p+1-\mu_{L}\right)\left(\frac{5}{4}-4\tau \left(\sigma^{*}\right)-\left(2-6\tau \left(\sigma^{*}\right)\right)\mu_{L}^{-}\right)=\frac{3p}{24-20p}$$

If $1\geq p\geq \frac{69-2\sqrt{534}}{25}$, aggregate welfare is

$$V^{T}=q\left(\mu_{L}p+1-\mu_{L}\right)\left(\frac{5}{4}-4\tau^{-}-\left(2-6\tau^{-}\right)\mu_{L}^{-}\right)=\frac{15p^{2}-294p+735}{128p+896}$$

where we used the fact that

$$\tau^{-}=\mu_{L}^{-}=\frac{p}{7+p}$$
